# Case Report: A Domestic Sponge Brush Used to Clean a Milk Feeding Bottle: The Source of Neonatal Meningitis Caused by *Pseudomonas aeruginosa*

**DOI:** 10.3389/fped.2021.725940

**Published:** 2021-09-23

**Authors:** Shinsuke Mizuno, Sayaka Matsuzaki, Koji Yokoyama, Keigo Hamahata, Akira Yoshida

**Affiliations:** Department of Pediatrics, Japanese Red Cross Wakayama Medical Center, Wakayama City, Japan

**Keywords:** neonatal meningitis, *Pseudomonas aeruginosa*, infection prevention, sponge brush, environment

## Abstract

*Pseudomonas aeruginosa* is a relatively rare cause of neonatal meningitis, and most patients have serious underlying diseases, prematurity, immunodeficiency, or anatomical abnormalities. We report the case of a 7-day-old girl with meningitis caused by *P. aeruginosa*. She was born full-term and had no immunodeficiency or anatomical abnormalities as far as our investigation ascertained. Through the use of anti-*Pseudomonas* antibiotics, she recovered without any complications other than a slight hearing disability revealed by audiology testing. *P. aeruginosa* was also isolated from a domestic sponge brush used to clean her milk bottle. Physicians should consider *P. aeruginosa* as a possible pathogen of neonatal meningitis even in full-term infants with no immunodeficiency or anatomical abnormalities. Physicians should give advice concerning appropriate hygiene practices to be applied to the neonate's environment.

## Introduction

Pathogens commonly causing neonatal bacterial meningitis in developed countries are Group B *Streptococcus* and *Escherichia coli*, followed by other Enterobacteriaceae such as *Klebsiella* spp. or *Enterobacter* spp. (1). *Pseudomonas aeruginosa* is a relatively rare pathogen, and most patients with meningitis caused by it have serious underlying diseases, prematurity, immunodeficiency, or anatomical abnormalities of the cribriform plate, inner ear, or congenital dermal sinus tract (2). Neurosurgical procedures also present a risk of meningitis caused by *P. Aeruginosa* (2). We report here a case of neonatal meningitis caused by *P. aeruginosa*. The case was born full-term and had no immunodeficiency or anatomical abnormalities according to our investigation. By testing her surrounding environment, a domestic sponge brush used to clean her milk bottle was considered as the most likely source of infection.

## Case Presentation

**Figure 1:**
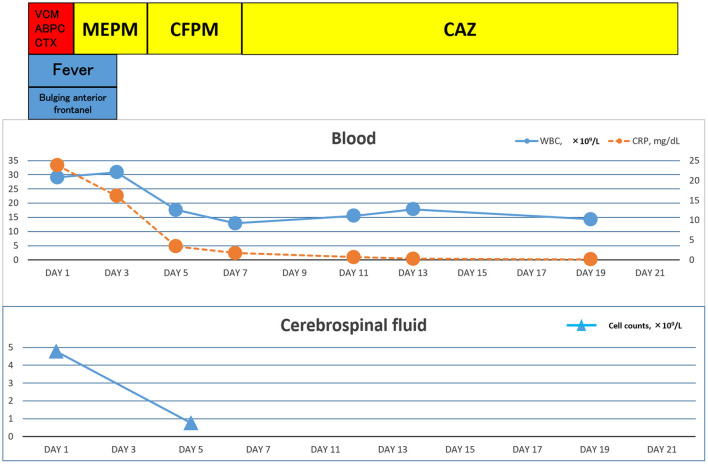
Clinical time course of the patient.

**Figure 2:**
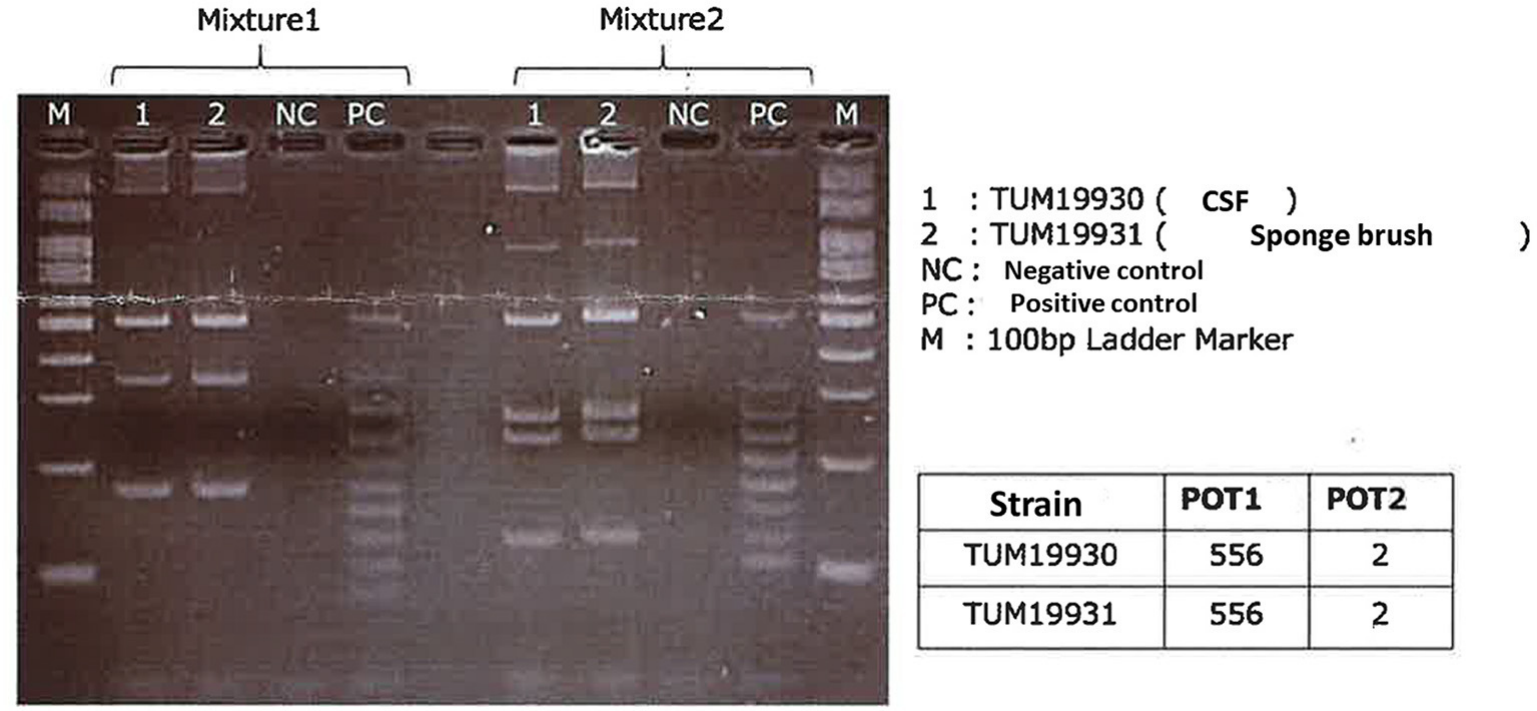
Agarose gel electrophoresis patterns of the PCR-based open reading frame typing (POT) using the Cica Geneus Pseudo POT KIT. PCR of reaction mixtures 1 and 2 was carried out for strain 1 and 2. Lane 1: strain 1 obtained from the patient's cerebrospinal fluid. Lane 2: strain 2 obtained from a cleaning sponge brush. The sizes of the bands in the M lane are 600, 500, 450, 400, 350, 300, 250, 200, 150, and 100 bp (from the top). PC, positive control; NC, negative control, used as an in-house ladder marker for detection PCR of *Pseudomonas aeruginosa*. Lines 1 and 2 showed the same band patterns, and the POT numbers of both strain 1 and 2 were the same values. The results from POT 1 and 2 values indicated these strains did not carry a metallo-beta-lactamase, such as IMP and VIM.

A 7-day-old girl was admitted to our hospital with a fever and poor feeding. Her family history was unremarkable. *Group B Streptococcus* was not detected in the vaginal and stool culture of her mother before delivery. She denied any exposure to toxins, drugs, tobacco, or alcohol. The neonate was delivered by vacuum assisted vaginal delivery, at the gestational age of 40 weeks and 3 days. Her birth weight was 3,436 g and Apgar score was 2/9. Before her birth, her heart rate was low, and suction delivery was performed because of shoulder dystocia. To alleviate her respiratory distress, non-invasive positive pressure ventilation was performed, and her respiratory condition immediately improved. She did not receive any antibiotics or parenteral nutrition. Auditory brainstem response testing revealed no abnormal findings at 4 days of age. She was discharged without any complications from hospital. No other neonates presented *P. aeruginosa* infection in the hospital around the same time as this patient. She was fed expressed breast milk in a bottle. Cleaning of the milk bottle with an appropriate sanitizer such as a hypochlorite-based disinfectant, sufficient rinsing, and drying after each use were not being done at home. She developed fever and failure to thrive at 7 days of age and was admitted to our hospital. On examination, she was febrile with a temperature of 38.2°C, tachycardiac and tachypneic, lethargic, and showing a bulging fontanelle. Her umbilical cord had already fallen off and she had simple dimple. No external malformations could be observed. On admission, initial investigations revealed an elevated white blood cell (WBC) count of 29.1 × 109 cells/L (normal range 3.8–10.0 × 109 cells/L) and C-reactive protein at 23.8 mg/L (normal range <2.0 mg/L). The coagulation screen, liver function tests, and renal function tests were normal. The cerebrospinal fluid (CSF) showed a markedly increased WBC count (4.7 × 109 /L), elevated protein level (15.9 g/L), and low glucose (1.6 mmol/L) when compared with an elevated serum glucose level (4.3 mmol/L). A Gram stain of CSF showed no organism on admission. Transfontanellar ultrasound performed on the admission day revealed no abnormal findings. Empirical therapy for neonatal meningitis with intravenous ampicillin and cefotaxime combined with acyclovir was initiated. On the next day, a CSF culture was positive for *P. aeruginosa*, and no pathogens were isolated by blood culture and urine culture. We stopped the empiric therapy regimen and started meropenem monotherapy. Once antimicrobial susceptibility test (Table 1) was confirmed, meropenem de-escalated to cefepime according to the Practical Guideline for Bacterial Meningitis 2014 in Japan. She developed agitation and myoclonus over the 3 days of cefepim treatment. We changed the antibiotic therapy to ceftazidime and continued for 3 weeks. We performed head MRI and whole body enhanced CT scans 12 days after admission. These images showed no findings of ventriculitis, hydrocephalus, brain abscess, or infarction. Structural defects of the cribriform plate, inner ear, or sinuses were also not detected. Her immunological status was assessed, and we found that the total absolute lymphocyte count, immunoglobulin levels, total complement measurement, lymphocyte analysis by flow cytometry, isohemagglutinins, response against lipopolysaccharide, and ability to produce reactive oxygen were all normal, except for slightly low immunoglobulin A level and natural killer cell count. Her clinical course was good, and she was discharged without any complications apart from a slight hearing disability for both ears detected on audiology testing. A brief summary has been shown in the timeline (shown in Figure 1). At the time of writing, 8 months after diagnosis, the patient is receiving outpatient follow-up without any problems. We investigated mother's breast skin, feeding bottle, breast pump milk collection kits, and related items as the source of infection. *Pseudomonas aeruginosa* was isolated from a cleaning sponge brush and had the same band pattern as the isolate obtained from CSF culture as shown by agarose gel electrophoresis patterns of the PCR-based open reading frame typing (POT) (shown in Figure 2).

**Table 1:** Antimicrobial susceptibility profile of the *Pseudomonas aeruginosa* isolated from CSF and sponge brush.

**Antimicrobial**	**MIC (mcg/ml)**	**Interpretation**
Piperacillin	<8	S
Piperacillin/tazobactam	<8	S
Ceftazidime	<4	S
Cefepime	<2	S
Aztreonam	<4	S
Imipenem	<1	S
Meropenem	<1	S
Gentamicin	<2	S
Amikacin	<8	S
Tobramycin	<2	S
Levofloxacin	<0.5	S
Ciprofloxacin	<0.5	S

*S, Susceptible*.

The study protocol was approved by the Institutional Review Board of the Japanese Red Cross Wakayama Medical Center (no. 855).

## Discussion

We learned the following two important lessons. Firstly, *P. aeruginosa* can cause neonatal meningitis even if the patient is delivered full-term and has no risk factors. Secondly, wet environments may be contaminated with the organism if appropriate preventative measures are not taken at home.

*Pseudomonas aeruginosa* is a rare pathogen causing neonatal meningitis, and most patients have risk factors including prematurity, immunodeficiency or anatomical abnormalities (1). *Pseudomonas aeruginosa* infections are related to phagocyte disorders, neutropenia, severe antibody deficiency, T cell immunodeficiency, or soft tissue injury (2). In this patient, there was no innate or acquired immune deficiency as far as we were able to determine. Recurrent bacterial infection did not occur in the 8-month outpatient follow-up period.

Wet environments can be a source of infection. Investigations of the epidemiology of neonatal intensive care unit outbreak cases indicated that *P. aeruginosa* can survive for a long time on the surfaces of wet environments (3). Domestic sponges are also considered to be potential sources of infection in older cases (4). In this patient's environment, the organism was detected on a sponge brush used to clean the baby's milk bottle, and it was considered to have the same origin as the one isolated from CSF according to agarose gel electrophoresis patterns of the PCR-based open reading frame typing (POT) study (5, 6). Appropriate cleaning and sanitation measures including washing equipment in detergent, rinsing, and drying after each use are recommended. For decontamination of sponge brushes, an atmospheric pressure steam method or a hypochlorite-based disinfectant method should be used where possible (7). It may be difficult to exclude other possible sources of infection because previous hospital sources and other home environment sources such as baby baths were not investigated.

In conclusion, *P. aeruginosa* can cause neonatal meningitis in patients without any risk factors, and wet environments can be a source of infection. We must be aware that *P. aeruginosa* can manifest as a community-acquired neonatal meningitis. Domestic wet environments should be managed by appropriate infection prevention measures. Further work should be done to determine whether routine disinfection of domestic wet environments may contribute to the prevention of *P. aeruginosa* neonatal meningitis.

## Data Availability Statement

The original contributions presented in the study are included in the article/supplementary material, further inquiries can be directed to the corresponding author/s.

## Ethics Statement

The studies involving human participants were reviewed and approved by the Institutional Review Board of the Japanese Red Cross Wakayama Medical Center (no. 855). Written informed consent to participate in this study was provided by the participants' legal guardian/next of kin.

## Author Contributions

SMi conceptualized and designed this study, drafted the initial version of this paper, and reviewed and revised this paper. SMa and KH designed the data collection instruments, collected the data, and reviewed and revised this paper. KY and AY interpreted the clinical data and critically reviewed this paper or important intellectual content. All authors approved the final version of this paper as submitted and agree to be accountable for all aspects of this work.

## Conflict of Interest

The authors declare that the research was conducted in the absence of any commercial or financial relationships that could be construed as a potential conflict of interest.

## Publisher's Note

All claims expressed in this article are solely those of the authors and do not necessarily represent those of their affiliated organizations, or those of the publisher, the editors and the reviewers. Any product that may be evaluated in this article, or claim that may be made by its manufacturer, is not guaranteed or endorsed by the publisher.
